# Why genes overlap in viruses

**DOI:** 10.1098/rspb.2010.1052

**Published:** 2010-07-07

**Authors:** Nicola Chirico, Alberto Vianelli, Robert Belshaw

**Affiliations:** 1 Department of Structural and Functional Biology, University of Insubria, Via JH Dunant 3, 21100 Varese, Italy; 2 Department of Zoology, University of Oxford, South Parks Road, Oxford OX1 3PS, UK

**Keywords:** genome compression, mutation, evolution, capsid, virus

## Abstract

The genomes of most virus species have overlapping genes—two or more proteins coded for by the same nucleotide sequence. Several explanations have been proposed for the evolution of this phenomenon, and we test these by comparing the amount of gene overlap in all known virus species. We conclude that gene overlap is unlikely to have evolved as a way of compressing the genome in response to the harmful effect of mutation because RNA viruses, despite having generally higher mutation rates, have less gene overlap on average than DNA viruses of comparable genome length. However, we do find a negative relationship between overlap proportion and genome length among viruses with icosahedral capsids, but not among those with other capsid types that we consider easier to enlarge in size. Our interpretation is that a physical constraint on genome length by the capsid has led to gene overlap evolving as a mechanism for producing more proteins from the same genome length. We consider that these patterns cannot be explained by other factors, namely the possible roles of overlap in transcription regulation, generating more divergent proteins and the relationship between gene length and genome length.

## 1. Introduction

Gene overlaps, which we define here as having nucleotides coding for more than one protein by being read in multiple reading frames, are a common feature of viruses. Proteins created by gene overlaps (sometimes called ‘overprinting’) are typically accessory proteins that play a role in viral pathogenicity or spread (Rancurel *et al*. 2009). These overlaps are typically assumed to be a form of genome compression, allowing the virus to increase its repertoire of proteins without increasing its genome length (Barrell *et al*. 1976; Scherbakov & Garber 2000; Lillo & Krakauer 2007; Chung *et al*. 2008).

Over the past several decades, many authors have suggested explanations for why gene overlap has arisen and become so common in viruses. In this study, we compare the amount of gene overlap across all known virus species to investigate the plausibility of these explanations. We find that the evidence is most consistent with the main effect being a physical constraint by the capsid (the protein capsule, into which the genome is packaged for transmission between host cells).

### (a) Mutation rate

RNA viruses make up approximately one-half of the 2000 known species of virus. They have an extremely high mutation rate and several authors have suggested that this could explain the evolution of gene overlap. The causality might be indirect: because most mutations are harmful, the high mutation rate will limit genome length, and thus new genes or gene regions must come from overlapping (Holmes 2009). Alternatively, several studies have shown how gene overlap in theory might mitigate the detrimental effects of mutation: (i) a numerical simulation (Belshaw *et al*. 2007) shows a benefit of overlapping except in the case of synergistic epistasis between fitness traits, which is rarely observed in RNA viruses (Elena *et al*. 2006), (ii) an analytical model (Peleg *et al*. 2004) quantifies the increasing harm of mutations in overlapping genomes in terms of the information cost (Krakauer 2000) and comes to the same conclusion: a fitness advantage of overlap when mutation rate is high, and (iii) overlapping genes can be seen as one of many ‘antiredundant’ mechanisms that may lead, in the case of mutation, to the damage of distinct functions simultaneously, and one which facilitates the removal of mutant genomes from the population (purging; Krakauer & Plotkin 2002). The authors show, through numerical simulations, that this kind of mechanism is likely to evolve in large populations, such as viruses. A more detailed study (Krakauer 2002) concludes that genome compression can increase the stability of the wild-type both by reducing mutation incidence (the advantage discussed above) and by reducing sequence redundancy.

This general argument predicts that DNA viruses, which make up the other half of the known virus species and which tend to have a lower mutation rate, will have less gene overlap.

### (b) Capsid structure

Gene overlap might have evolved if genome length is physically limited by the size of the capsid. This was suggested over 30 years ago (Fiddes 1977) and has been invoked since to explain individual gene overlaps (Bransom *et al*. 1995). Some observations are consistent with capsid size constraining genome length: in most viruses studied, it is not possible to package an artificially enlarged genome (Cann 2001; Campbell 2007), and many studies on different virus groups have found virus genome length to be positively correlated with capsid size (Belyi & Muthukumar 2006; Nurmemmedov *et al*. 2007; Hu *et al*. 2008; Krupovic & Bamford 2008; Luque *et al*. 2009; Zandi & Van der Schoot 2009). Furthermore, the hypothesis is testable because, as we describe below, some capsid types might be expected to constrain genome length more than others (Cavalier-Smith 1983; Casjens 1985).

**Figure 1.**
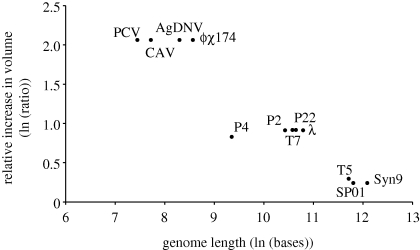
Predicted capsid volume increases in moving up to the nearest available *T* number compared with genome length for some DNA viruses. For further details see §2.

Many viruses have capsids that are icosahedral (20 sided), varying in the number of protein units (capsomers) that form each side. In such viruses, an increase in capsid size is generally achieved through increases in the number rather than the size of these capsomers (Rossmann & Erickson 1985; Chapman & Liljas 2003; Shepherd & Reddy 2005; Krupovic & Bamford 2008). These increases in capsomer number are in discrete steps following a geometric pattern represented by the so-called *T* (Triangulation) number series (Caspar & Klug 1962), which appears to be thermodynamically determined (Zandi *et al*. 2004). As the *T* values increase, the differences in volume (as a percentage) between adjacent *T* numbers become smaller, with the product of the capsid diameter and the reciprocal of the square root of *T* remaining constant (Walker & Anderson 1970; Rossmann & Erickson 1985; Hu *et al*. 2008). The actual pattern of historical transitions between different *T* numbers is unknown and probably determined by the type of fold found in the capsomer (Ahlquist 2005; Bamford *et al*. 2005; Krupovic & Bamford 2008). We discuss this in the electronic supplementary material. In figure 1, we illustrate the general principle using some DNA viruses, chosen because both their *T* number is known, and because of capsomer fold similarity, we can infer the likely next highest possible *T* number.

The critical point is that a small virus with an icosahedral capsid, unlike a large virus with an icosahedral capsid, cannot physically make relatively minor adjustments to the size of its capsid by increasing its *T* number. We speculate that such viruses are therefore more likely to acquire novel gene function via overlap. The situation is different among viruses with non-icosahedral capsids because in theory adjustments in capsid size, and hence genome length, are as easy to make for small viruses as large ones, e.g. individual capsomers can simply be added onto the end of a helical capsid, as shown for M13 phage (Cann 2001) and Tobacco Mosaic Virus (Dawson *et al*. 1989). We therefore predict that the negative relationship between gene overlap and genome length known at least for RNA viruses (Belshaw *et al*. 2007) will be stronger among viruses with icosahedral capsids than among viruses with non-icosahedral capsids.

### (c) Gene length

The negative relationship between overlap proportion and genome length mentioned above could merely be an artefact of a relationship between gene length and genome length, and hence not require any biological explanation. The length of RNA virus genes with replicase functions increases with genome length (Belshaw *et al*. 2008); indeed, there may be a general tendency for genes to become larger in taxa with larger genomes, e.g. eukaryotes tend to have longer genes than prokaryotes (Rost 2002). It appears that most gene overlaps in RNA viruses started from two originally contiguous genes (Belshaw *et al*. 2007), so if mean gene length increases with genome length, then there will be fewer opportunities for overlaps to evolve in a given length of nucleotides. We investigate the importance of this relationship.

### (d) Expression regulation

Some gene overlaps may have evolved to couple gene expression rather than to compress the genome (Normark *et al*. 1983; Krakauer 2000). This is thought to be common in bacteria (Johnson & Chisholm 2004; Lillo & Krakauer 2007), and there are a few possible examples in viruses (Scherbakov & Garber 2000), e.g. in the RNA phage MS2 the start of the lysis gene overlaps with the end of the coat gene; translation of the lysis gene requires coat protein synthesis termination followed by reinitiation (Berkhout *et al*. 1987), the frequency of which may be a mechanism to regulate relative protein levels. Suspected cases of gene regulation typically involve short terminal overlaps, so we explored the effect of excluding from our analyses all overlaps of length less than 60 bases, which is approximately the minimum size of a functional protein (Neidigh *et al*. 2002).

## 2. Material and methods

### (a) Virus genomes

We cannot use measures of overlap in individual species as data for statistical tests because many species will have the same overlap between homologous genes. Such overlaps will often have a common origin and hence individual species do not represent independent data. However, we can use virus families as independent data because there is very little homology across families and thus most gene overlaps are likely to have been independently acquired. For example, in RNA viruses the only putative homology across all families is a small region of their replicase (Zanotto *et al*. 1996). We therefore use family means throughout. We follow the taxonomy of the NCBI Virus Genome website (‘GenBank’; currently at http://www.ncbi.nlm.nih.gov/genomes/GenomesHome.cgi?taxid=10239), and our analysis was based on a downloaded RefSeq flat file of July 2008. Unassigned genera and species were treated as additional independent data points. We follow NCBI's placement of Hepadnaviridae and Caulimoviridae among the RNA viruses despite their mature virion containing DNA. This is sensible owing to their possession of reverse transcriptase, which clearly allies them to reverse-transcribing RNA viruses.

We measured gene overlap only as the overlap between the reading frames of different genes, i.e. we ignored regulatory regions (which are often unknown) or overlaps in the same frame. We also only included genomes that are classified by NCBI as either reviewed or validated (see below for exceptions). In the case of segmented viruses, we only included species where all segments were thus classified. The taxa included and excluded in our analysis, along with mean overlap proportion and genome length, are listed in the electronic supplementary material, table S1. We also added the recently discovered overlaps in Potyviridae (Chung *et al*. 2008), Reoviridae (Firth 2008; Firth & Atkins 2008*b*) and Sequiviridae (Firth & Atkins 2008*a*), and the overlap in Schizochytrium single-stranded RNA virus (Takao *et al*. 2006). We were thus able to calculate means of gene overlap for 36 RNA and 26 DNA virus families (or unassigned genera or species). Exclusion of the less well-annotated species is important, e.g. their inclusion obscures the underlying relationship between overlap proportion and genome length among double-stranded (ds) DNA viruses (electronic supplementary material, figure S1). Excluded data represent seven families plus 10 unassigned genera or species of DNA virus, and 10 families plus 21 unassigned genera or species of RNA virus.

### (b) Statistics

For analysing the trends in overlap proportion between families, we excluded families that have no gene overlap and then used natural logarithms of the overlap proportion and genome length to obtain approximate linear relationships. This exclusion is necessary for logarithmic transformation and should not affect our findings: only one DNA virus family, the Nanoviridae, has no overlap (as discussed above, we are only considering families represented by at least one validated or reviewed RefSeq entry). This family is unusual in having multiple small circular segments each coding for a single protein. Eight RNA virus families are also excluded because they appear to have no gene overlap. Some of these probably do have some gene overlap: some Bunyaviridae provisional RefSeq entries have gene overlap, and an overlapping gene has been reported in a member of the Picornaviridae—Theiler's murine encephalomyelitis virus (Theilovirus; Van Eyll & Michiels 2000) and in a member of the Dicistroviridae (Sabath *et al*. 2009). In order to compare the amount of gene overlap in RNA and DNA viruses, we calculated the mean of the family means, both including and excluding the small number of families that lacked overlap.

### (c) Mutation rate

We included all the estimated mutation rates that we could find in the literature (electronic supplementary material, table S3). Rates are typically expressed as the number of substitutions per base per round of genome copying. A few studies give the rate per round of cell infection, which will be higher but not misleadingly so, especially given the likely error margins on these values. Despite an extensive search, we could find estimates for only six DNA viruses, only two of which have genome lengths within the range of RNA viruses.

### (d) Capsid type

Using a standard reference work (Van Regenmortel *et al*. 2000), we classified virus families (and unassigned genera and species) as having either *icosahedral* or *flexible* capsid types. We treat as flexible all non-icosahedral types, e.g. capsids described as spherical, filamentous, helical or rod-shaped, or where there is no well-defined capsid (electronic supplementary material, table S1).

### (e) Relationship between changes in capsid volume and genome length

The discrete changes in the size of icosahedral capsids are relatively larger for viruses with small genomes than for viruses with large genomes. We illustrate this principle using some DNA viruses whose *T* number is known and where the next highest *T* number can be predicted from other viruses that share the same capsomer fold (see electronic supplementary material, Supplementary Methods and table S2). These include eight tailed, icosahedral dsDNA bacteriophages with the HK97 fold and similar capsid molecular weight (around 40 kDa). To expand the range of *T* values, we included four single-stranded (ss) DNA viruses with *T* = 1 (no dsDNA viruses with this property are known). These viruses have a different fold, β-barrel fold, but their coat protein is of similar weight. The only transition that is hypothetical is from *T* = 16 (SPO1 and Syn9) to *T* = 19, i.e. no dsDNA phage with *T* = 19 is known, and we predicted this transition from the ‘3*n* + 1 rule’ (Thuman-Commike *et al*. 1999). For these calculations, the capsid has been approximated to a sphere (Purohit *et al*. 2005) and the relative volume increase has been calculated assuming the product of the radius and the reciprocal of the square root of *T* to be constant (Walker & Anderson 1970; Rossmann & Erickson 1985; Hu *et al*. 2008).

## 3. Results

**Figure 2.**
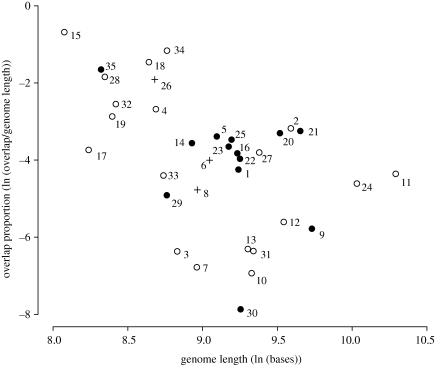
Relationship between overlap proportion (the proportion of the genome that is within an overlap) and total genome length for RNA virus families, both expressed as natural logarithms. Points are means for the following taxa, all of which have at least one well-curated genome and some gene overlap. Open circles are families with icosahedral capsids; closed circles have flexible capsids; crosses are families with indeterminate capsid forms. Linear regression, *r*^2^ = 0.24, *p* = 0.003. (1) Arenaviridae (*n* = 2); (2) Arteriviridae (*n* = 3); (3) Astroviridae (*n* = 4); (4) Birnaviridae (*n* = 3); (5) Bornaviridae (*n* = 1); (6) Bromoviridae (*n* = 9); (7) Caliciviridae (*n* = 9); (8) Caulimoviridae (*n* = 7); (9) Closteroviridae (*n* = 7); (10) Comoviridae (*n* = 7); (11) Coronaviridae (*n* = 9); (12) Cystoviridae (*n* = 3); (13) Flaviviridae (*n* = 26); (14) Flexiviridae (*n* = 22); (15) Hepadnaviridae (*n* = 1); (16) Hordeivirus (*n* = 1); (17) Leviviridae (*n* = 6); (18) Luteoviridae (*n* = 8); (19) Nodaviridae (*n* = 2); (20) Orthomyxoviridae (*n* = 1); (21) Paramyxoviridae (*n* = 3); (22) Pecluvirus (*n* = 1); (23) Potyviridae (*n* = 60); (24) Reoviridae (*n* = 16); (25) Retroviridae (*n* = 11); (26) Schizochytrium single-stranded RNA virus (*n* = 1); (27) Sclerophthora macrospora virus A (*n* = 1); (28) Sequiviridae (*n* = 2); (29) Sobemovirus (*n* = 9); (30) Tobamovirus (*n* = 6); (31) Tobravirus (*n* = 1); (32) Togaviridae (*n* = 9); (33) Tombusviridae (*n* = 9); (34) Totiviridae (*n* = 3); (35) Tymoviridae (*n* = 5); (36) Umbravirus (*n* = 2).

**Figure 3.**
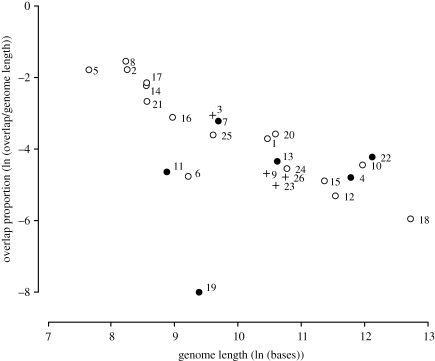
Relationship between overlap proportion and total genome length for DNA virus families. See figure 2 legend for explanation of symbols. Linear regression *r*^2^ = 0.39, *p* < 0.001. (1) Adenoviridae (*n* = 12); (2) Anellovirus (*n* = 1); (3) Bacillus phage GIL16c (*n* = 1); (4) Baculoviridae (*n* = 1); (5) Circoviridae (*n* = 3); (6) Corticovirus (*n* = 1); (7) Fuselloviridae (*n* = 3); (8) Geminiviridae (*n* = 82); (9) Geobacillus phage GBSV1 (*n* = 1); (10) Herpesviridae (*n* = 26); (11) Inoviridae (*n* = 18); (12) Iridoviridae (*n* = 1); (13) Lipothrixviridae (*n* = 2); (14) Microviridae (*n* = 13); (15) Myoviridae (*n* = 35); (16) Papillomaviridae (*n* = 13); (17) Parvoviridae (*n* = 8); (18) Phycodnaviridae (*n* = 1); (19) Plasmaviridae (*n* = 1); (20) Podoviridae (*n* = 32); (21) Polyomaviridae (*n* = 2); (22) Poxviridae (*n* = 7); (23) Salmonella phage ST64B (*n* = 1); (24) Siphoviridae (*n* = 106); (25) Tectiviridae (*n* = 1); (26) Xanthomonas phage OP2 (*n* = 1). The outlier represents the four base pair overlap in Acholeplasma phage L2 (RefSeq NC_001447).

We find that 75 per cent of the approximately 2000 known virus species have at least some gene overlap. The negative relationship between overlap proportion and genome length in RNA viruses (figure 2; linear regression *r*^2^ = 0.23, *p* = 0.006) reported previously (Belshaw *et al*. 2007) also exists among DNA viruses (figure 3; linear regression, *r*^2^ = 0.38, *p* = 0.002). This relationship is found within all the constituent virus groups, e.g. ssDNA and dsDNA viruses (electronic supplementary material, figures S2 and S3), and within the two types of overlap: *internal overlaps*, where one gene is completely overlapped by a larger second, and *terminal overlaps*, where two genes overlap for part of their lengths—one upstream and one downstream (electronic supplementary material, figures S4 and S5).

### (a) Mutation rate

**Figure 4.**
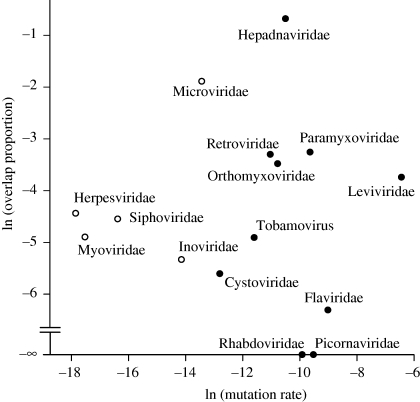
Relationship between overlap proportion and mutation rate. Open circles are family means for DNA viruses, closed circles are family means for RNA viruses. The negative infinity value represents families without overlap (all values are logarithmically transformed).

As summarized in figure 4 (electronic supplementary material, table S3), the mutation rates of RNA viruses are higher than those of DNA viruses. An important caveat here is that some DNA viruses have a longer genome than any RNA virus (compare figures 2 and 3) and are thus not comparable. There are estimates for only two DNA viruses of similar genome length to RNA viruses (Inoviridae and Microviridae). Nevertheless, these two values are lower than the mutation rate of any known RNA virus (we have found two lower mutation rate estimates for RNA viruses, in MLV and FLUBV, but these are both questionable because there are much higher estimates for the same or related viruses).

Mutation rate appears to be a poor explanation of gene overlap in viruses because RNA viruses, despite their higher mutation rate, tend to have less genome overlap than DNA viruses of similar genome length—the opposite of the expectation if mutation causes overlap. The mean number of nucleotides in an overlap as a percentage of the genome length is 4.6 per cent for RNA viruses and 6.6 per cent for DNA viruses whose genome length is within the upper limit for RNA viruses (and 4.3% for DNA viruses if we include all genome lengths). Excluding families that lack overlap from these calculations does not change this result: the value is unchanged for small DNA viruses but increases to 6.1 per cent for RNA viruses and to 4.5 per cent for DNA viruses of all genome lengths. A lower value for overlap proportion in RNA viruses published previously by one of us (Belshaw *et al*. 2007) was an error.

As shown in figure 4, there is no obvious relationship between overlap proportion and mutation rate (linear regression, *p* = 0.50, *n* = 13). Introducing stepwise into an ANOVA (i) viral type (DNA or RNA) and (ii) genome length did not reveal significant interactions or a significant overall increase in the relationship between overlap and mutation (*p* > 0.26). The same outcome was obtained using—rather than a logarithmic—an arcsin transformation, which linearizes less effectively but allows inclusion of zero overlap values (*n* = 15).

### (b) Capsid structure

Separating virus families into ones with icosahedral capsids and those with flexible capsids (figures 2 and 3) shows that this negative relationship between overlap and genome length is actually derived only from the former group. Combining RNA and DNA viruses into one regression analysis, the relationship between overlap proportion and genome length is strong among families with icosahedral capsids (linear regression, *r*^2^ = 0.27, *p* = 0.001, *n* = 35) but there is no evidence for it among families with flexible capsids (linear regression, *r*^2^ = 0.02, *p* = 0.58, *n* = 19). We find the same result treating RNA and DNA viruses separately: in RNA viruses with icosahedral capsids *r*^2^ = 0.26 and *p* = 0.02, while in RNA viruses with flexible capsids *r*^2^ = 0.14 and *p* = 0.21; in DNA viruses with icosahedral capsids *r*^2^ = 0.81 and *p* < 0.001, while in DNA viruses with flexible capsids *r*^2^ = 0.08 and *p* = 0.60.

This finding is not caused by the sample size differences. For both virus types, we sampled families with icosahedral capsids to give us the same number as families with flexible capsids and repeated the analysis 100 000 times: in only 9 per cent (RNA viruses) or 0.1 per cent (DNA viruses) of the replicates did the relationship decline to the same level as among families with flexible capsids, as measured by their *p*-value (excluding the outlying value for Plasmaviridae raises the DNA virus result only to 0.5%). As the data for RNA and DNA viruses are independent, these two probabilities (9% and 0.1%) can simply be multiplied.

### (c) Gene length

Most of the decrease in overlap proportion with increasing genome length is caused by overlaps becoming rarer rather than shorter. The evidence for this is that there is a strong negative relationship between the number of overlaps per unit length and genome length (linear regression, *r*^2^ = 0.37 and 0.38 in RNA and DNA viruses, respectively; electronic supplementary material, figure S6). By contrast, there is a much weaker negative relationship between mean overlap length and genome length (linear regression, *r*^2^ = 0.02 and 0.16 in RNA and DNA viruses, respectively; electronic supplementary material, figure S7).

We are confident that this rarity of gene overlaps in larger viruses is not an artefact caused by larger viruses having longer genes, and hence fewer genes per unit length. This is because there is a very highly significant negative relationship between (i) the ratio of overlap number to gene number and (ii) genome length (linear regression, *r*^2^ = 0.21 and *p* = 0.007 in RNA viruses, *r*^2^ = 0.42 and *p* < 0.001 in DNA viruses; electronic supplementary material, figure S8).

### (d) Expression regulation

We find that the relationship between overlap proportion and genome length is strengthened by the removal of short overlaps (electronic supplementary material, figure S9; *r*^2^ values increase to 0.33 and 0.71 for RNA and DNA viruses, respectively). This is consistent with most large gene overlaps having evolved as a form of genome compression but with some short terminal overlaps having evolved to regulate gene expression (and others perhaps being neutral).

## 4. Discussion

Our findings are consistent with the inflexibility of icosahedral capsids constraining virus genome length, and gene overlap being a mechanism for acquiring new gene functions under this constraint.

We do not find evidence for gene overlap having evolved as a response to the deleterious effects of mutation. However, we clearly need mutation rate estimates for more small DNA viruses, especially dsDNA viruses and those ssDNA viruses that are known to have a high rate of evolution (substitutions per site per year; Duffy *et al*. 2008). Also, the expectation of more overlap among RNA compared with DNA viruses, owing to their higher mutation rates, could be masked by RNA viruses having a secondary structure (Yoffe *et al*. 2008) and this further constraining overlap at synonymous sites (Krakauer 2000).

The hypothesis that gene overlap evolved in response to length constraint by the capsid assumes that there is a fitness cost to the virus in enlarging its capsid. We think this is a reasonable assumption because capsid enlargement can be expected to reduce the virus's reproductive output, which we can measure as the burst size or growth rate. Many studies have shown burst size/growth rate to be limited by the host cell's resources (Eigen *et al*. 1991; Hadas *et al*. 1997; You *et al*. 2002; Kim & Yin 2004) and producing a larger capsid will require more of these resources.

We think it very unlikely that the inevitable misannotations in some RefSeq entries will have affected our conclusions. We recognize that detecting gene overlaps is difficult, with new overlaps recently being discovered using specially designed computer programs (Shibuya & Rigoutsos 2002; Firth & Brown 2006; Sabath *et al*. 2008). However, the two critical points for our study are, first, that the trends we report are also found among small genomes (RNA viruses and the smaller DNA viruses) as well as among the larger DNA viruses, and it is among the latter where we expect most misannotations (because many overlaps will not have been confirmed experimentally); second, misannotation will be a source of random error that will obscure trends rather than create them, e.g. in our study the inclusion of provisional RefSeq genomes obscures completely the relationship between overlap proportion and genome length in dsDNA viruses (electronic supplementary material, figure S1); indeed, our study is a good example of the importance of data quality in comparative genomic studies. Similarly, uncertainty over the location of start codons might mean that short terminal overlaps are more likely to be misannotations than other overlaps; however, exclusion of these only strengthens the support for our findings (electronic supplementary material, figure S9), and we find the same relationship between overlap proportion and genome length when we consider only internal overlaps (electronic supplementary material, figures S4 and S5), which are generally unlikely to be misannotations because on average they are much larger (Belshaw *et al*. 2007).

The relationship between overlap porportion and genome length is much weaker among icosahedral RNA compared with icosahedral DNA viruses (*r*^2^ values of 0.26 and 0.81, respectively). We suggest the following explanation. There is some evidence among RNA viruses that the genome length can physically influence the size of the capsid (Schneemann 2006; Hu *et al*. 2008), perhaps because RNA virus genomes tend to be involved in capsid formation (Hohn 1976; Prasad & Prevelige 2003; Rao 2006); by contrast, the genome of DNA viruses enters the already formed (pro)capsid (Fane & Prevelige 2003; Prasad & Prevelige 2003). RNA viruses might thereby have a little more flexibility in expanding their genome length without overlap.

This putative lower level of constraint among RNA viruses is consistent with some other observations. First, polyploidy (the packaging of more than one genome or genome segment copy) is known among RNA but not DNA viruses. Three of these examples are in families with flexible capsids (Orthomyxoviridae, Paramyxoviridae and Retroviridae) and one is in a family with icosahedral capsids, Birnaviridae (Rager *et al*. 2002; Luque *et al*. 2009). Second, most of the known examples of species being polymorphic for capsid size (so-called *T* number modulation) are among RNA viruses (Krol *et al*. 1999; Rao 2006; Schneemann 2006; Zandi & Van der Schoot 2009) rather than DNA viruses (Baker *et al*. 1999).

Another observation is consistent with capsid size influencing genome architecture. Many RNA virus species have segmented genomes (analogous to chromosomes), and a few of these package these genomic segments into separate capsids. It has been suggested (Agranovsky 1996) that this phenomenon evolved in order to relieve packaging constraints and the need for overlap. For example, one can compare some members of the Comoviridae and Bromoviridae, which package genomic segments into separate icosahedral capsids and in which there is little or no overlap, with Tymoviridae, which have single icosahedral capsids and make extensive use of overlap. These are all plant viruses whose capsid size might be severely constrained by the need to pass through the plasmodesmata (Lucas & Gilbertson 1994; Rao 2006). Genome segmentation is rare among DNA viruses, but two of the three families in which it occurs, Nanovirus and Geminiviridae, are also icosahedral plant viruses in which at least some members have genomic segments that are packaged separately (the third family is the non-icosahedral Polydnaviridae).

There are of course other factors involved in the evolution of gene overlaps in viruses, e.g. there are overlaps in non-icosahedral viruses and in other organisms: (i) selection for faster replication could lead to gene overlap: if the small genome of some viruses is the result of selection for faster replication (small genomes being quicker to copy), then we might expect small viruses to have more overlap because overlap allows more proteins to be coded for by a given genome length. There is a more general evolutionary model to explain genome compression which incorporates mutation rate, population size and replication rate (Krakauer 2002), but our comparative data cannot test this model. Selection for replication speed has also been proposed as an explanation for the high mutation rate of RNA viruses, via a possible trade-off between copying speed and copying fidelity (Elena & Sanjuan 2005; Belshaw *et al*. 2008), and this area deserves more attention, (ii) proteins and protein regions created de novo by overlapping may have novel chemical and physical properties. This is suggested both by contemplating the effects of a resulting shift in codon usage bias (Keese & Gibbs 1992) and by observing that such genes tend to have unusual protein structure and composition (Rancurel *et al*. 2009). Nevertheless, we find it difficult to explain the striking relationship between overlap and genome length if overlaps evolved primarily in order to create proteins with novel chemical or physical properties—why should this be more important for small viruses than for large viruses? Also, we do not know the relative importance of gene overlap in creating the genomes of today's RNA and DNA viruses. Large dsDNA viruses have acquired many genes via gene duplication and horizontal transfer from the host (Shackelton & Holmes 2004), while there are very few examples of these processes known among RNA viruses (Holmes 2009); however, the comparison needs to be between RNA viruses and DNA of comparable genome length, and this has yet to be done, and (iii) some overlaps may be selectively neutral. It is likely that overlaps evolved from the translation of novel or extended open reading frames (ORFs) created by the mutational gain or loss of start and stop codons, respectively, although the actual molecular mechanisms involved are varied (Belshaw *et al*. 2007). Subsequent mutations could lengthen these ORFs and new functions could be acquired gradually by the novel polypeptides. Thus, initial short overlaps may be essentially neutral (Lillo & Krakauer 2007). We need to test this model by reconstructing the appearance and changes in length of overlaps on viral phylogenies, which could be combined with examining changes in capsid structure, e.g. we predict that short overlaps precede longer ones and increases in capsid size follow gene acquisition.

There has been a search for universal factors that influence an organism's genome length (Lynch & Conery 2003; Charlesworth & Barton 2004; Cavalier-Smith 2005), and—beyond a commonly observed genome reduction in symbionts—there is little consensus at the moment. We believe that our analysis of one possible form of genome compression points to a taxon-specific factor, namely the capsid, and casts doubt on the role of a more general phenomenon, namely mutation. We also believe that our study is a good example of contingency in evolution: natural selection acted on capsids favouring the icosahedral structure because of its stability and economical design, the consequence of which was a fixed volume of the interior. This led to the proliferation of overlaps not necessarily as the best possible solution to increase the genome coding capacity, but as the only one possible. Where this constraint is less stringent, less overlap is present. In effect, the capsid poses an engineering problem for the creation of genomic novelty, and gene overlap is the way around it (Maynard Smith 1986).

## References

[RSPB20101052C1] Agranovsky A. A. 1996 The principles of molecular organization, expression and evolution of closteroviruses: over the barriers. Adv. Virus Res. 47, 119–158 10.1016/S0065-3527(08)60735-6 (doi:10.1016/S0065-3527(08)60735-6) 8895832 PMC7130501

[RSPB20101052C2] Ahlquist P. 2005 Virus evolution: fitting lifestyles to a T. Curr. Biol. 15, R465–R467 10.1016/j.cub.2005.06.016 (doi:10.1016/j.cub.2005.06.016) 15964268

[RSPB20101052C3] Baker T. S. Olson N. H. Fuller S. D. 1999 Adding the third dimension to virus life cycles: three-dimensional reconstruction of icosahedral viruses from cryo-electron micrographs. Microbiol. Mol. Biol. Rev. 63, 862–922 10585969 10.1128/mmbr.63.4.862-922.1999 PMC98980

[RSPB20101052C4] Bamford D. H. Grimes J. M. Stuart D. I. 2005 What does structure tell us about virus evolution? Curr. Opin. Struct. Biol. 15, 655–663 10.1016/j.sbi.2005.10.012 (doi:10.1016/j.sbi.2005.10.012) 16271469

[RSPB20101052C5] Barrell B. G. Air G. M. Hutchison C. A. 1976 Overlapping genes in bacteriophage-φ*χ*174. Nature 264, 34–41 10.1038/264034a0 (doi:10.1038/264034a0) 1004533

[RSPB20101052C6] Belshaw R. Pybus O. G. Rambaut A. 2007 The evolution of genome compression and genomic novelty in RNA viruses. Genome Res. 17, 1496–1504 10.1101/gr.6305707 (doi:10.1101/gr.6305707) 17785537 PMC1987338

[RSPB20101052C7] Belshaw R. Gardner A. Rambaut A. Pybus O. G. 2008 Pacing a small cage: mutation and RNA viruses. Trends Ecol. Evol. 23, 188–193 10.1016/j.tree.2007.11.010 (doi:10.1016/j.tree.2007.11.010) 18295930 PMC7125972

[RSPB20101052C8] Belyi V. A. Muthukumar M. 2006 Electrostatic origin of the genome packing in viruses. Proc. Natl Acad. Sci. USA 103, 17 174–17 178 10.1073/pnas.0608311103 (doi:10.1073/pnas.0608311103) PMC1859905 17090672

[RSPB20101052C9] Berkhout B. Schmidt B. F. Van Strien A. Van Boom J. Van Westrenen J. Van Duin J. 1987 Lysis gene of bacteriophage MS2 is activated by translation termination at the overlapping coat gene. J. Mol. Biol. 195, 517–524 10.1016/0022-2836(87)90180-X (doi:10.1016/0022-2836(87)90180-X) 3656424

[RSPB20101052C10] Bransom K. L. Weiland J. J. Tsai C. H. Dreher T. W. 1995 Coding density of the Turnip Yellow Mosaic Virus genome: roles of the overlapping coat protein and p206-readthrough coding regions. Virology 206, 403–412 10.1016/S0042-6822(95)80056-5 (doi:10.1016/S0042-6822(95)80056-5) 7831796

[RSPB20101052C11] Campbell A. 2007 Bacteriophages. In Fields virology, *vol. 1* (eds Knipe D. M. Howley P. M.), pp. 769–791 Philadelphia, PA: Lippincott, Williams & Wilkins

[RSPB20101052C12] Cann A. 2001 Principles of molecular virology. New York, NY: Academic Press

[RSPB20101052C13] Casjens S. 1985 Nucleic acid packaging by viruses. In Virus structure and assembly (ed. Casjens S.), pp. 75–147 Boston, MA: Jones and Bartlett

[RSPB20101052C14] Caspar D. L. D. Klug A. 1962 Physical principles in the construction of regular viruses. Cold Spring Harb. Symp. Quant. Biol. 27, 1–24 14019094 10.1101/sqb.1962.027.001.005

[RSPB20101052C15] Cavalier-Smith T. 1983 Genetic symbionts and the origin of split genes and linear chromosomes. In Endocytobiology II: intracellular space as oligogenetic ecosystem (eds Schenk H. E. A. Schwemmler W.), pp. 29–45 Berlin, Germany: de Gruyter

[RSPB20101052C16] Cavalier-Smith T. 2005 Economy, speed and size matter: evolutionary forces driving nuclear genome miniaturization and expansion. Ann. Bot. 95, 147–175 10.1093/aob/mci010 (doi:10.1093/aob/mci010) 15596464 PMC4246715

[RSPB20101052C17] Chapman M. S. Liljas L. 2003 Structural folds of viral proteins. Adv. Protein Chem. 64, 125–196 10.1016/S0065-3233(03)01004-0 (doi:10.1016/S0065-3233(03)01004-0) 13677047

[RSPB20101052C18] Charlesworth B. Barton N. 2004 Genome size: does bigger mean worse? Curr. Biol. 14, R233–R235 10.1016/j.cub.2004.02.054 (doi:10.1016/j.cub.2004.02.054) 15043833

[RSPB20101052C19] Chung B. Y. W. Miller W. A. Atkins J. F. Firth A. E. 2008 An overlapping essential gene in the Potyviridae. Proc. Natl Acad. Sci. USA 105, 5897–5902 10.1073/pnas.0800468105 (doi:10.1073/pnas.0800468105) 18408156 PMC2311343

[RSPB20101052C20] Dawson W. O. Lewandowski D. J. Hilf M. E. Bubrick P. Raffo A. J. Shaw J. J. Grantham G. L. Desjardins P. R. 1989 A Tobacco Mosaic Virus-hybrid expresses and loses an added gene. Virology 172, 285–292 10.1016/0042-6822(89)90130-X (doi:10.1016/0042-6822(89)90130-X) 2773319

[RSPB20101052C21] Duffy S. Shackelton L. A. Holmes E. C. 2008 Rates of evolutionary change in viruses: patterns and determinants. Nat. Rev. Genet. 9, 267–276 10.1038/nrg2323 (doi:10.1038/nrg2323) 18319742

[RSPB20101052C22] Eigen M. Biebricher C. K. Gebinoga M. Gardiner W. C. 1991 The hypercycle. Coupling of RNA and protein biosynthesis in the infection cycle of an RNA bacteriophage. Biochemistry 30, 11 005–11 018 10.1021/bi00110a001 (doi:10.1021/bi00110a001) 1932025

[RSPB20101052C23] Elena S. F. Sanjuan R. 2005 Adaptive value of high mutation rates of RNA viruses: separating causes from consequences. J. Virol. 79, 11 555–11 558 10.1128/JVI.79.18.11555-11558.2005 (doi:10.1128/JVI.79.18.11555-11558.2005) PMC1212614 16140732

[RSPB20101052C24] Elena S. F. Carrasco P. Daros J. A. Sanjuan R. 2006 Mechanisms of genetic robustness in RNA viruses. EMBO Rep. 7, 168–173 10.1038/sj.embor.7400636 (doi:10.1038/sj.embor.7400636) 16452927 PMC1369264

[RSPB20101052C25] Fane B. A. Prevelige P. E. 2003 Mechanism of scaffolding-assisted viral assembly. Adv. Protein Chem. 64, 259–299 10.1016/S0065-3233(03)01007-6 (doi:10.1016/S0065-3233(03)01007-6) 13677050

[RSPB20101052C26] Fiddes J. C. 1977 The nucleotide sequence of a viral DNA. Sci. Am. 237, 54–67 10.1038/scientificamerican1277-54 (doi:10.1038/scientificamerican1277-54) 929160

[RSPB20101052C27] Firth A. E. 2008 Bioinformatic analysis suggests that the Orbivirus VP6 cistron encodes an overlapping gene. Virol. J. 5, 48 10.1186/1743-422X-5-48 (doi:10.1186/1743-422X-5-48) 18489030 PMC2373779

[RSPB20101052C28] Firth A. E. Atkins J. F. 2008a Bioinformatic analysis suggests that a conserved ORF in the waikaviruses encodes an overlapping gene. Arch. Virol. 153, 1379–1383 10.1007/s00705-008-0119-5 (doi:10.1007/s00705-008-0119-5) 18535758

[RSPB20101052C29] Firth A. E. Atkins J. F. 2008b Bioinformatic analysis suggests that the Cypovirus 1 major core protein cistron harbours an overlapping gene. Virol. J. 5, 62 10.1186/1743-422X-5-62 (doi:10.1186/1743-422X-5-62) 18492230 PMC2409309

[RSPB20101052C30] Firth A. E. Brown C. M. 2006 Detecting overlapping coding sequences in virus genomes. BMC Bioinform. 7, 75 10.1186/1471-2105-7-75 (doi:10.1186/1471-2105-7-75) PMC1395342 16483358

[RSPB20101052C31] Hadas H. Einav M. Fishov I. Zaritsky A. 1997 Bacteriophage T4 development depends on the physiology of its host *Escherichia coli*. Microbiology 143, 179–185 10.1099/00221287-143-1-179 (doi:10.1099/00221287-143-1-179) 9025292

[RSPB20101052C32] Hohn T. 1976 Packaging of genomes in bacteriophages: a comparison of ssRNA bacteriophages and dsDNA bacteriophages. Proc. R. Soc. Lond. B 276, 143–150 10.1098/rstb.1976.0105 (doi:10.1098/rstb.1976.0105) 13425

[RSPB20101052C33] Holmes E. C. 2009 The evolution and emergence of RNA viruses. Oxford, UK: Oxford University Press

[RSPB20101052C34] Hu Y. Zandi R. Anavitarte A. Knobler C. M. Gelbart W. M. 2008 Packaging of a polymer by a viral capsid: the interplay between polymer length and capsid size. Biophys. J. 94, 1428–1436 10.1529/biophysj.107.117473 (doi:10.1529/biophysj.107.117473) 17981893 PMC2212672

[RSPB20101052C35] Johnson Z. I. Chisholm S. W. 2004 Properties of overlapping genes are conserved across microbial genomes. Genome Res. 14, 2268–2272 10.1101/gr.2433104 (doi:10.1101/gr.2433104) 15520290 PMC525685

[RSPB20101052C36] Keese P. K. Gibbs A. 1992 Origins of genes: ‘big bang’ or continuous creation. Proc. Natl Acad. Sci. USA 89, 9489–9493 1329098 10.1073/pnas.89.20.9489 PMC50157

[RSPB20101052C37] Kim H. Yin J. 2004 Energy-efficient growth of phage Qβ in *Escherichia coli*. Biotechnol. Bioeng. 88, 148–156 10.1002/bit.20226 (doi:10.1002/bit.20226) 15449299

[RSPB20101052C38] Krakauer D. C. 2000 Stability and evolution of overlapping genes. Evolution 54, 731–739 10937248 10.1111/j.0014-3820.2000.tb00075.x

[RSPB20101052C39] Krakauer D. C. 2002 Evolutionary principles of genomic compression. Comments Theor. Biol. 7, 215–236 10.1080/08948550214053 (doi:10.1080/08948550214053)

[RSPB20101052C40] Krakauer D. C. Plotkin J. B. 2002 Redundancy, antiredundancy, and the robustness of genomes. Proc. Natl Acad. Sci. USA 99, 1405–1409 10.1073/pnas.032668599 (doi:10.1073/pnas.032668599) 11818563 PMC122203

[RSPB20101052C41] Krol M. A. Olson N. H. Tate J. Johnson J. E. Baker T. S. Ahlquist P. 1999 RNA-controlled polymorphism in the *in vivo* assembly of 180-subunit and 120-subunit virions from a single capsid protein. Proc. Natl Acad. Sci. USA 96, 13 650–13 655 10.1073/pnas.96.24.13650 (doi:10.1073/pnas.96.24.13650) PMC24119 10570127

[RSPB20101052C42] Krupovic M. Bamford D. H. 2008 Virus evolution: how far does the double beta-barrel viral lineage extend? Nat. Rev. Microbiol. 6, 941–948 10.1038/nrmicro2033 (doi:10.1038/nrmicro2033) 19008892

[RSPB20101052C43] Lillo F. Krakauer D. C. 2007 A statistical analysis of the three-fold evolution of genomic compression through frame overlaps in prokaryotes. Biol. Direct 2, 22 10.1186/1745-6150-2-22 (doi:10.1186/1745-6150-2-22) 17877818 PMC2174442

[RSPB20101052C44] Lucas W. J. Gilbertson R. L. 1994 Plasmodesmata in relation to viral movement within leaf tissue. Annu. Rev. Phytopathol. 32, 387–411 10.1146/annurev.py.32.090194.002131 (doi:10.1146/annurev.py.32.090194.002131)

[RSPB20101052C45] Luque D. Rivas G. Alfonso C. Carrascosa J. L. Rodriguez J. F. Caston J. R. 2009 Infectious bursal disease virus is an icosahedral polyploid dsRNA virus. Proc. Natl Acad. Sci. USA 106, 2148–2152 10.1073/pnas.0808498106 (doi:10.1073/pnas.0808498106) 19164552 PMC2650107

[RSPB20101052C46] Lynch M. Conery J. S. 2003 The origins of genome complexity. Science 302, 1401–1404 10.1126/science.1089370 (doi:10.1126/science.1089370) 14631042

[RSPB20101052C47] Maynard Smith J. 1986 The problems of biology. Oxford, UK: Oxford University Press

[RSPB20101052C48] Neidigh J. W. Fesinmeyer R. M. Andersen N. H. 2002 Designing a 20-residue protein. Nat. Struct. Biol. 9, 425–430 10.1038/nsb798 (doi:10.1038/nsb798) 11979279

[RSPB20101052C49] Normark S. Bergstrom S. Edlund T. Grundstrom T. Jaurin B. Lindberg F. P. Olsson O. 1983 Overlapping genes. Annu. Rev. Genet. 17, 499–525 10.1146/annurev.ge.17.120183.002435 (doi:10.1146/annurev.ge.17.120183.002435) 6198955

[RSPB20101052C50] Nurmemmedov E. Castelnovo M. Catalano C. E. Evilevitch A. 2007 Biophysics of viral infectivity: matching genome length with capsid size. Q. Rev. Biophys. 40, 327–356 18423102 10.1017/S0033583508004666

[RSPB20101052C51] Peleg O. Kirzhner V. Trifonov E. Bolshoy A. 2004 Overlapping messages and survivability. J. Mol. Evol. 59, 520–527 10.1007/s00239-004-2644-5 (doi:10.1007/s00239-004-2644-5) 15638463

[RSPB20101052C52] Prasad B. V. V. Prevelige P. E. 2003 Viral genome organization. Adv. Protein Chem. 64, 219–258 10.1016/S0065-3233(03)01006-4 (doi:10.1016/S0065-3233(03)01006-4) 13677049

[RSPB20101052C53] Purohit P. K. Inamdar M. M. Grayson P. D. Squires T. M. Kondev J. Phillips R. 2005 Forces during bacteriophage DNA packaging and ejection. Biophys. J. 88, 851–866 10.1529/biophysj.104.047134 (doi:10.1529/biophysj.104.047134) 15556983 PMC1305160

[RSPB20101052C54] Rager M. Vongpunsawad S. Duprex W. P. Cattaneo R. 2002 Polyploid measles virus with hexameric genome length. EMBO J. 21, 2364–2372 10.1093/emboj/21.10.2364 (doi:10.1093/emboj/21.10.2364) 12006489 PMC126007

[RSPB20101052C55] Rancurel C. Khosravi M. Dunker K. Romero P. Karlin D. 2009 Overlapping genes produce proteins with unusual sequence properties and offer insight into de novo protein creation. J. Virol. 83, 10 719–10 736 10.1128/JVI.00595-09 (doi:10.1128/JVI.00595-09) PMC2753099 19640978

[RSPB20101052C56] Rao A. L. N. 2006 Genome packaging by spherical plant RNA viruses. Annu. Rev. Phytopathol. 44, 61–87 10.1146/annurev.phyto.44.070505.143334 (doi:10.1146/annurev.phyto.44.070505.143334) 16480335

[RSPB20101052C57] Rossmann M. Erickson J. 1985 Structure and assembly of icosahedral shells. In Virus structure and assembly (ed. Casjens S.), pp. 29–73 Boston, MA: Jones and Bartlett

[RSPB20101052C58] Rost B. 2002 Did evolution leap to create the protein universe? Curr. Opin. Struct. Biol. 12, 409–416 10.1016/S0959-440X(02)00337-8 (doi:10.1016/S0959-440X(02)00337-8) 12127462

[RSPB20101052C59] Sabath N. Landan G. Graur D. 2008 A method for the simultaneous estimation of selection intensities in overlapping genes. PLoS ONE 3, e3996 10.1371/journal.pone.0003996 (doi:10.1371/journal.pone.0003996) 19098983 PMC2601044

[RSPB20101052C60] Sabath N. Price N. Graur D. 2009 A potentially novel overlapping gene in the genomes of Israeli acute paralysis virus and its relatives. Virol. J. 6, 144 10.1186/1743-422X-6-144 (doi:10.1186/1743-422X-6-144) 19761605 PMC2754452

[RSPB20101052C61] Scherbakov D. V. Garber M. B. 2000 Overlapping genes in bacterial and phage genomes. Mol. Biol. 34, 485–495 10.1007/BF02759558 (doi:10.1007/BF02759558) 11042850

[RSPB20101052C62] Schneemann A. 2006 The structural and functional role of RNA in icosahedral virus assembly. Annu. Rev. Microbiol. 60, 51–67 10.1146/annurev.micro.60.080805.142304 (doi:10.1146/annurev.micro.60.080805.142304) 16704342

[RSPB20101052C63] Shackelton L. A. Holmes E. C. 2004 The evolution of large DNA viruses: combining genomic information of viruses and their hosts. Trends Microbiol. 12, 458–465 10.1016/j.tim.2004.08.005 (doi:10.1016/j.tim.2004.08.005) 15381195

[RSPB20101052C64] Shepherd C. M. Reddy V. S. 2005 Extent of protein–protein interactions and quasi-equivalence in viral capsids. Proteins 58, 472–477 10.1002/prot.20311 (doi:10.1002/prot.20311) 15558545

[RSPB20101052C65] Shibuya T. Rigoutsos I. 2002 Dictionary-driven prokaryotic gene finding. Nucleic Acids Res. 30, 2710–2725 10.1093/nar/gkf338 (doi:10.1093/nar/gkf338) 12060689 PMC117281

[RSPB20101052C66] Takao Y. Mise K. Nagasaki K. Okuno T. Honda D. 2006 Complete nucleotide sequence and genome organization of a single-stranded RNA virus infecting the marine fungoid protist Schizochytrium sp. J. Gen. Virol. 87, 723–733 10.1099/vir.0.81204-0 (doi:10.1099/vir.0.81204-0) 16476996

[RSPB20101052C67] Thuman-Commike P. A. Greene B. Malinski J. A. Burbea M. McGough A. Chiu W. Prevelige P. E. 1999 Mechanism of scaffolding-directed virus assembly suggested by comparison of scaffolding-containing and scaffolding-lacking P22 procapsids. Biophys. J. 76, 3267–3277 10354452 10.1016/S0006-3495(99)77479-5 PMC1300296

[RSPB20101052C68] Van Eyll O. Michiels T. 2000 Influence of the Theiler's virus L* protein on macrophage infection, viral persistence, and neurovirulence. J. Virol. 74, 9071–9077 10.1128/JVI.74.19.9071-9077.2000 (doi:10.1128/JVI.74.19.9071-9077.2000) 10982352 PMC102104

[RSPB20101052C69] Van Regenmortel M. H. V. 2000 Virus taxonomy: classification and nomenclature of viruses. San Diego, CA: Academic Press

[RSPB20101052C70] Walker D. H. Jr Anderson T. F. 1970 Morphological variants of coliphage P1. J. Virol. 5, 765–782 4193834 10.1128/jvi.5.6.765-782.1970 PMC376070

[RSPB20101052C71] Yoffe A. M. Prinsen P. Gopal A. Knobler C. M. Gelbart W. M. Ben-Shaul A. 2008 Predicting the sizes of large RNA molecules. Proc. Natl Acad. Sci. USA 105, 16 153–16 158 10.1073/pnas.0808089105 (doi:10.1073/pnas.0808089105) PMC2570976 18845685

[RSPB20101052C72] You L. C. Suthers P. F. Yin J. 2002 Effects of *Escherichia coli* physiology on growth of phage T7 *in vivo* and *in silico*. J. Bacteriol. 184, 1888–1894 10.1128/JB.184.7.1888-1894.2002 (doi:10.1128/JB.184.7.1888-1894.2002) 11889095 PMC134924

[RSPB20101052C73] Zandi R. Van der Schoot P. 2009 Size regulation of ss-RNA viruses. Biophys. J. 96, 9–20 10.1529/biophysj.108.137489 (doi:10.1529/biophysj.108.137489) 18931258 PMC2710049

[RSPB20101052C74] Zandi R. Reguera D. Bruinsma R. F. Gelbart W. M. Rudnick J. 2004 Origin of icosahedral symmetry in viruses. Proc. Natl Acad. Sci. USA 101, 15 556–15 560 10.1073/pnas.0405844101 (doi:10.1073/pnas.0405844101) PMC524849 15486087

[RSPB20101052C75] Zanotto P. M. D. Gibbs M. J. Gould E. A. Holmes E. C. 1996 A reevaluation of the higher taxonomy of viruses based on RNA polymerases. J. Virol. 70, 6083–6096 8709232 10.1128/jvi.70.9.6083-6096.1996 PMC190630

